# Choroidal Osteoma Complicated by Choroidal Neovascularization: A Case Report

**DOI:** 10.7759/cureus.100540

**Published:** 2025-12-31

**Authors:** Aisha Ahmed Khan, Zaid Azhar, Muhammad Amer Awan

**Affiliations:** 1 Ophthalmology, Shifa International Hospital Islamabad, Islamabad, PAK; 2 Ophthalmology, Shifa Tameer-E-Millat University Shifa College of Medicine, Islamabad, PAK

**Keywords:** anti-vegf treatment, calcification, choroid, choroidal neovascularization, choroidal osteoma, intravitreal injections, swept source oct (ss-oct)

## Abstract

A 13-year-old girl with no prior systemic or ocular comorbidities presented to the eye clinic at Shifa International Hospital, Islamabad, Pakistan, with complaints of blurred vision in her left eye. Fundus examination revealed a well-defined, yellow-orange lesion at the posterior pole. B-scan ultrasonography demonstrated an echo-dense lesion, most prominent at low gain, while swept source optical coherence tomography (SS-OCT) showed a hyperreflective choroidal mass with associated subretinal fluid (SRF). Based on these findings, a diagnosis of choroidal osteoma complicated by choroidal neovascularization (CNV) was established. This case report describes the management of a pediatric case of CNV related to choroidal osteoma with a series of intravitreal anti-vascular endothelial growth factor (anti-VEGF) injections over nine months, resulting in significant regression of CNV and a reduction in SRF. She continues to be monitored with multimodal imaging and intermittent therapy, demonstrating favorable anatomical and functional outcomes.

## Introduction

Choroidal osteomas were initially reported by Gass et al. in the year 1978 [1]. It is defined as an exceptional benign bony tumor that occurs in the choroid, which looks like a raised yellowish-orange growth with clear edges present under the retinal pigment epithelium (RPE) with fine blood vessels crossing over it in the macular region of the fundus [1,2]. It is known to happen mainly in young females and to affect one eye only in 75% of cases [3]. The most frequent complication of choroidal osteoma is choroidal neovascularization (CNV), followed by subretinal fluid, bleeding, and exudative or hemorrhagic retinal detachment [4]. Pediatric cases of choroidal osteomas are significant to add to the literature as they are rare and potentially sight-threatening in children, leading to blinding amblyopia or, in rare cases, serous or hemorrhagic retinal detachment as a consequence of CNV. The modalities used to diagnose choroidal osteomas are optical coherence tomography (OCT), ultrasonography (B-scan), fundus fluorescent angiography (FFA), indocyanine green fluorescence angiography (ICGA), and OCT angiography (OCTA) [5]. We describe a case of choroidal osteoma with associated CNV in a 13-year-old girl who was treated with different anti-VEGF injections to achieve improved clinical and visual outcomes.

## Case presentation

A 13-year-old girl presented to the Ophthalmology Clinic at Shifa International Hospital, Islamabad, Pakistan, with a history of painless, sudden-onset blurry vision in her left eye for several days. She had no history of refractive error, ocular trauma, surgery, or systemic/ocular comorbidities. Family history was negative for retinal disorders. On examination, best-corrected visual acuity (VA) was 6/6 in the right eye and 6/15 in the left eye. Anterior segment examination of both eyes was unremarkable.

**Figure 1:**
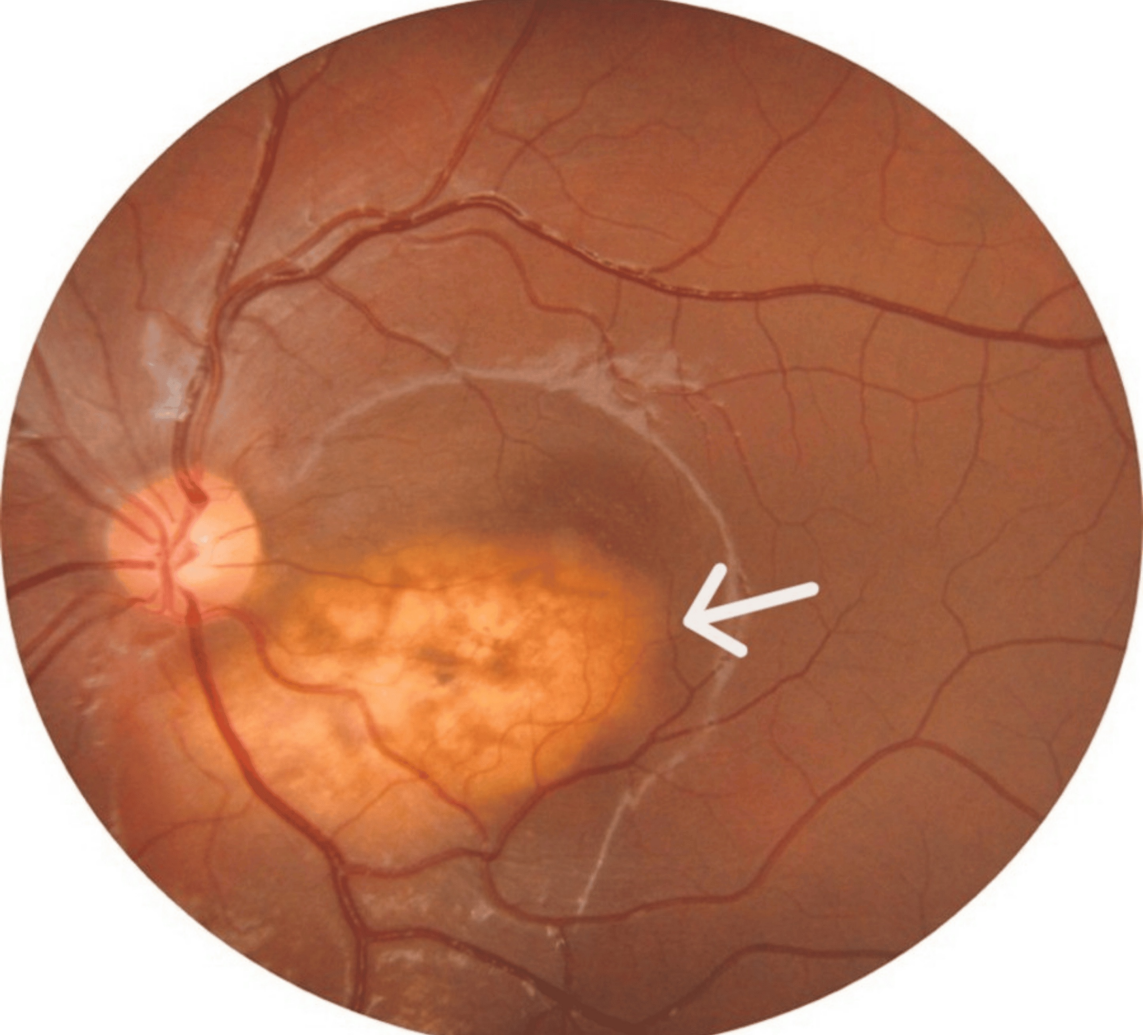
Fundus photo showing the elevated yellow-orange sub-retinal lesion along infero-temporal vascular arcade with partial macular involvement and RPE pigment changes. White arrow pointing to the lesion RPE: retinal pigment epithelium

**Figure 2:**
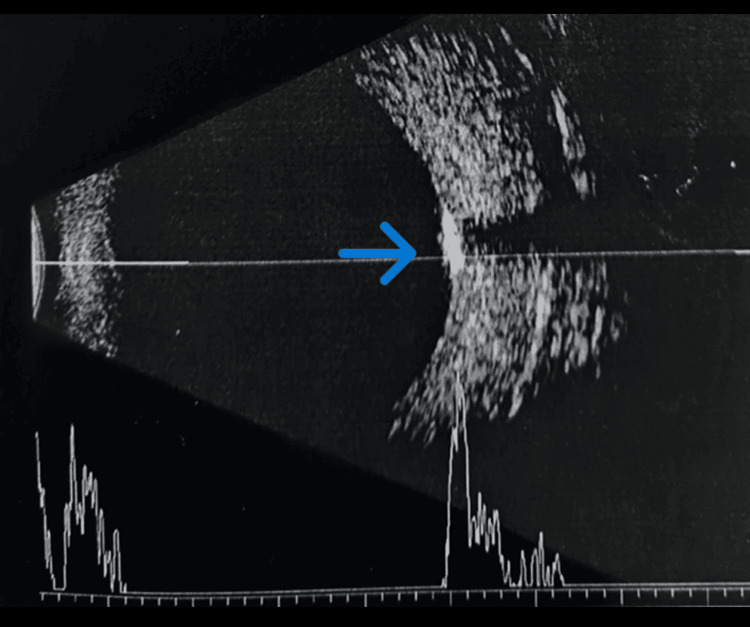
B-scan demonstrating an echo-dense lesion with a hypo-dense cone-shaped shadow behind it. Blue arrow pointing to the lesion

**Figure 3:**
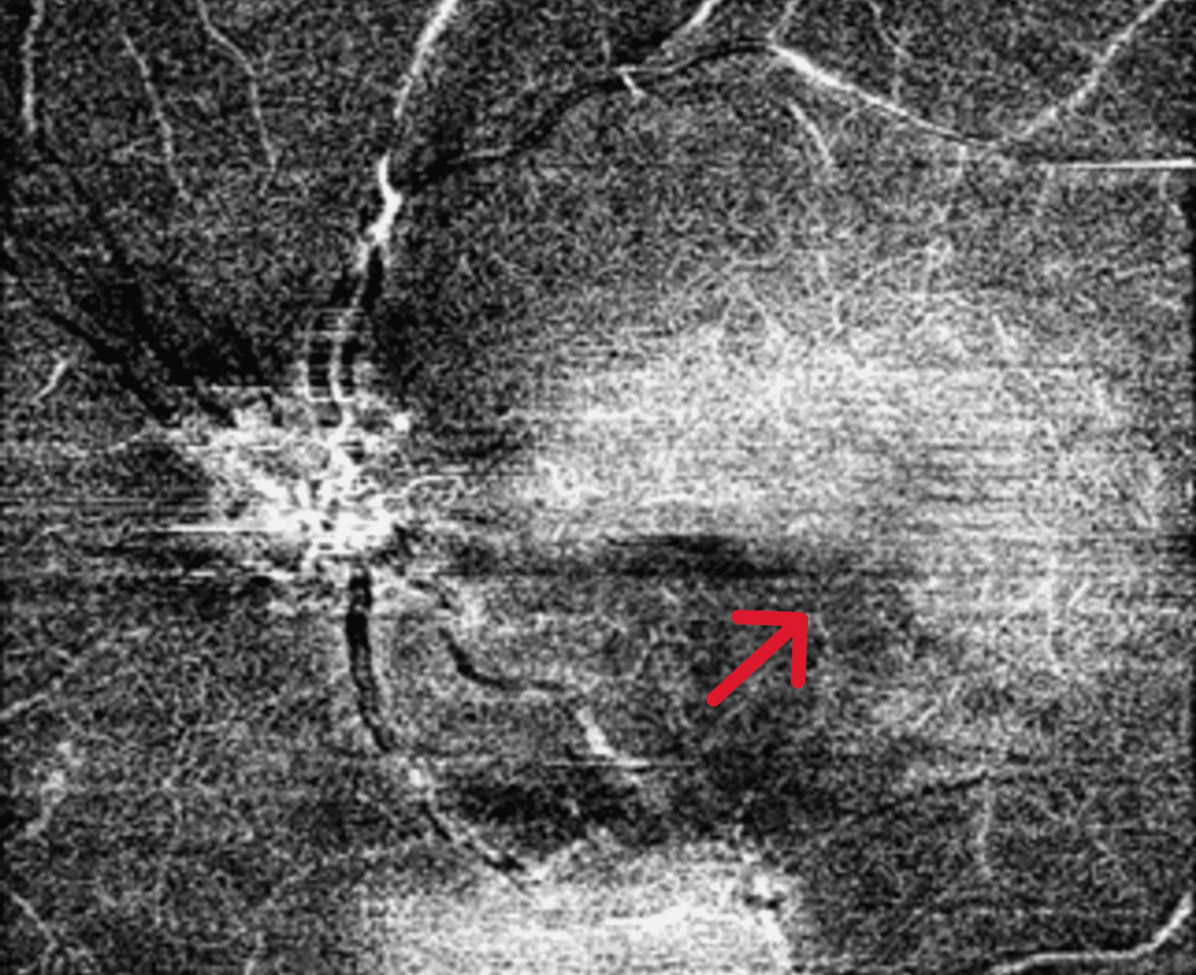
OCT-A image showing CNV in the superficial layer of the choriocapillaris, as demonstrated by the red arrow. CNV: choroidal neovascularization; OCT-A: optical coherence tomography angiography

**Figure 4:**
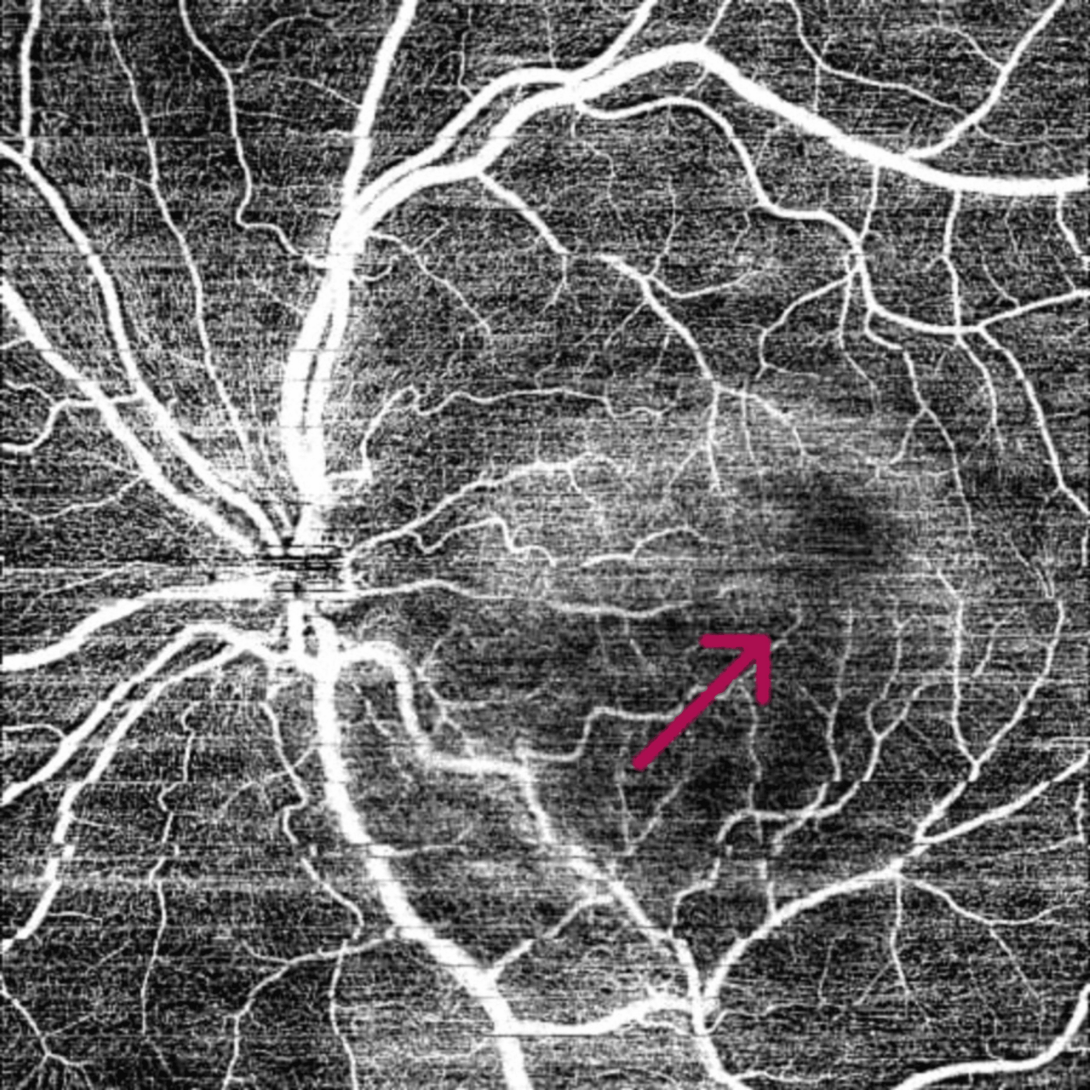
OCT-A image showing CNV in the deep layer of the choriocapillaris, as demonstrated by the red arrow. CNV: choroidal neovascularization; OCT-A: optical coherence tomography angiography

**Figure 5:**
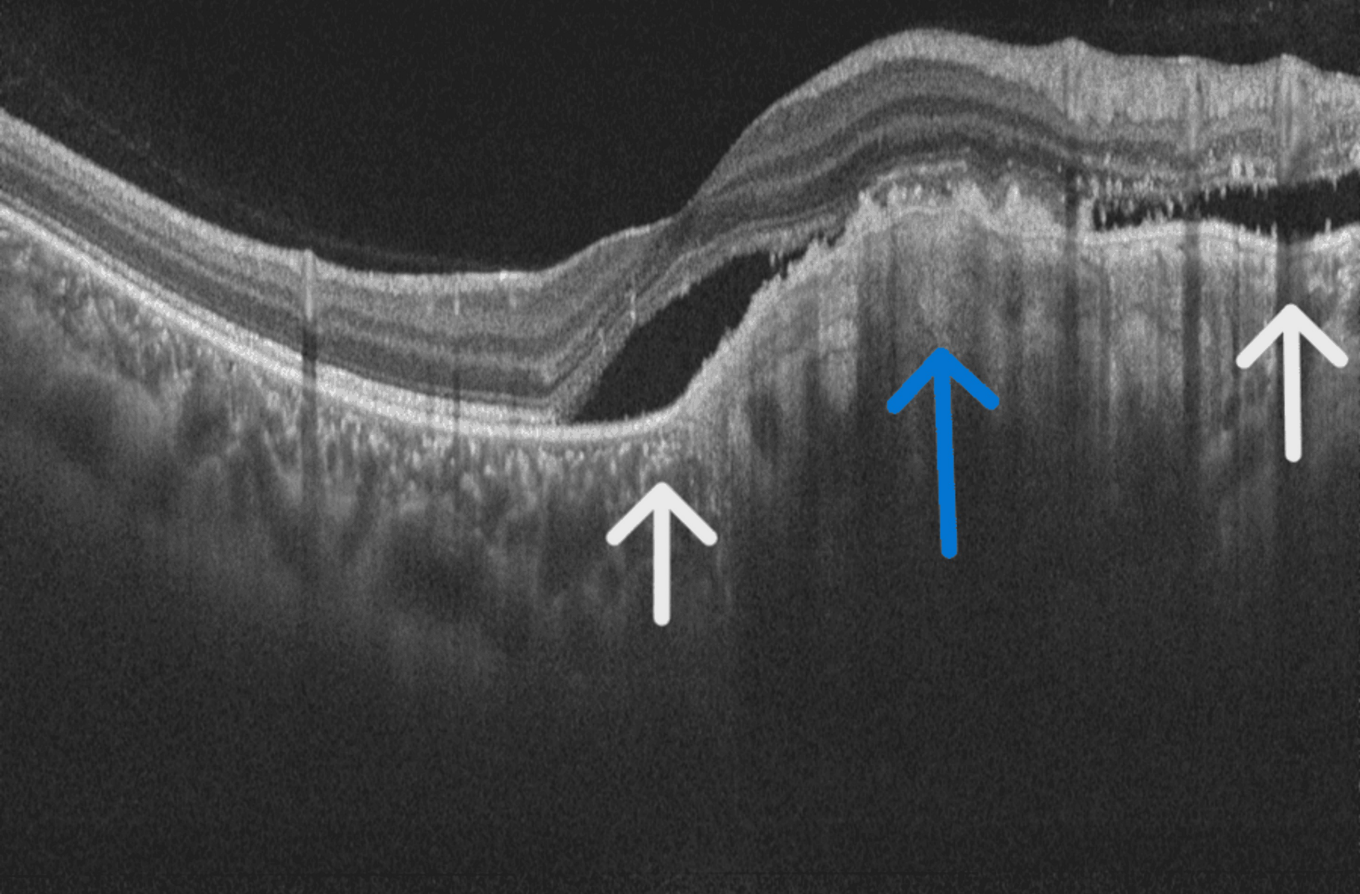
OCT of the left eye showing clear vitreo-retinal interface, normal retinal layers with significant disruption of the IS-OS junction with sub-retinal fluid and a sub-RPE hyper-reflective mass arising from the choroid causing RPE detachment with RPE structural alteration and a hyper-reflective layer over the lesion giving a tuft-like appearance. White arrow pointing to sub-retinal fluid and blue arrow pointing to the sub-RPE hyper-reflective mass. OCT: optical coherence tomography; RPE: retinal pigment epithelium

Fundus examination of the left eye revealed a well-defined, elevated, yellow-orange subretinal lesion measuring 5-6-disc diameters along the inferotemporal vascular arcade, with partial macular involvement and RPE alterations (Figure 1). B-scan ultrasonography demonstrated a hyperechoic lesion with a distinct hypoechoic cone-shaped shadow posteriorly, best visualized at low gain (Figure 2). OCTA also demonstrated the presence of CNV (Figures 3-4). SS-OCT revealed an elevated, hyperreflective sub-RPE lesion arising from the choroid, associated with subretinal fluid, IS/OS junction disruption, and RPE irregularities (Figure 5). A number of differentials, such as retinoblastoma, phthisis bulbi, calcified drusen, dystrophic calcification, acute or resolving sub-retinal hemorrhage or infection, metastatic calcification, amelanotic or atypical malignant melanoma, metastatic carcinoma, leukemic or lymphomatous infiltrates, choroidal hemangioma, and macular choroidal scars were considered. Since our patient was healthy with no positive history of any illnesses or trauma, with a normal family history, a diagnosis of choroidal osteoma with CNV was established.

**Figure 6:**
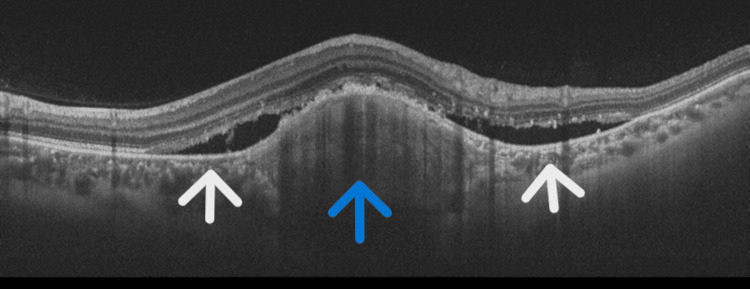
OCT showing a significant reduction in sub-retinal fluid following intravitreal anti-VEGF. Blue arrow pointing to the sub-RPE hyper-reflective mass and white arrows pointing to sub-retinal fluid. OCT: optical coherence tomography; RPE: retinal pigment epithelium

Relevant systemic investigations, including serum creatinine, C-reactive protein, angiotensin-converting enzyme levels, antinuclear antibodies, and Quantiferon-TB Gold, were within normal limits. After taking informed consent from the patient's guardian, the patient was started on monthly intravitreal anti-VEGF therapy. She initially received two intravitreal bevacizumab injections (1.25 mg/0.05 mL each) and one ranibizumab injection (0.5 mg/0.05 mL) over three months, resulting in improvement of VA to 6/9 in the left eye and reduction of subretinal fluid on OCT (Figure 6).

**Figure 7:**
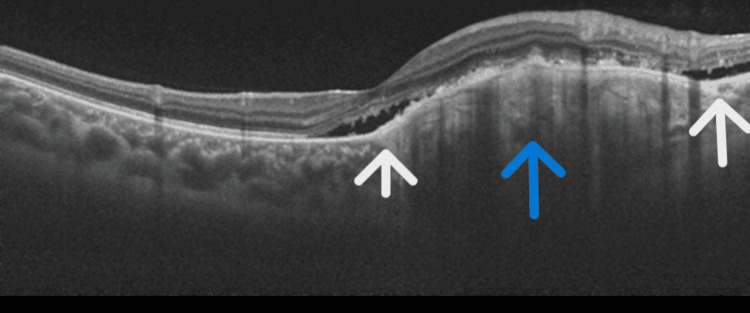
OCT showing increase in sub-retinal fluid. Blue arrow pointing to a sub-RPE hyper-reflective mass and white arrows pointing to sub-retinal fluid. OCT: optical coherence tomography; RPE: retinal pigment epithelium

Due to the unavailability of bevacizumab and ranibizumab, the patient subsequently received three monthly intravitreal aflibercept injections (2 mg/0.05 mL). At the three-month review, VA remained stable at 6/6 in the right eye and 6/9 in the left eye; however, OCT showed increased subretinal fluid (Figure 7). Given her prior favorable response, the patient was advised to resume monthly bevacizumab injections. She continues to be monitored with serial VA assessments, dilated fundus examinations, OCT imaging, and ongoing intravitreal therapy.

## Discussion

Choroidal osteomas are rare, benign tumors characterized by the presence of mature bone tissue within an otherwise healthy choroid. Their exact etiology remains unclear. In the largest case series to date, Shields et al. [6] reported on 74 eyes and found that the condition predominantly affects women, with CNV occurring in approximately 30% of cases. To date, a total of 85 cases of secondary CNV associated with choroidal osteomas have been reported in the literature [7].

Shields et al. [6] reported that choroidal osteomas show progression in approximately 51% of eyes, with up to 50% undergoing decalcification within a decade, a process often associated with poor visual prognosis. The disease typically demonstrates a slow and unpredictable course, with CNV sometimes developing as late as nine years after the initial diagnosis [5]. Decalcification is commonly linked to RPE alterations, loss of choriocapillaris, and degeneration of photoreceptors, ultimately leading to visual decline [8]. A characteristic feature of choroidal osteomas on imaging is the presence of an extensive surface vascular network, often appearing as vascular tufts. [9] In our case, similar findings were observed, including an irregular choriocapillaris layer beneath areas of RPE disruption, with associated IS-OS junction damage and loss. The unique finding in this case is that the patient had no systemic diseases, no active or chronic conditions, and no history of trauma that could have led to choroidal calcification.

Decalcification, with subsequent retinal RPE damage and alterations in the choriocapillaris, has been postulated to disturb normal retinal homeostasis and trigger VEGF synthesis [10]. This mechanism is considered the primary driver of CNV as a chronic complication of choroidal osteoma, typically evidenced by the presence of subretinal fluid [7]. Such findings were also demonstrated in our case.

The treatment of CNV associated with subretinal fluid demonstrates the best response with intravitreal anti-VEGF therapy [11]. Papastefanou et al. [7] reported favorable outcomes with intravitreal bevacizumab, showing a reduction in central retinal thickness and marked resolution of subretinal fluid in eyes with CNV secondary to choroidal osteoma. Similarly, Ahmadieh et al. [12] documented successful use of bevacizumab, while Sarıgül Sezenöz et al. [13] reported effective treatment with ranibizumab. More recently, aflibercept has also shown promising results in managing CNV in choroidal osteomas [14]. Although no head-to-head comparative studies exist specifically for CNV secondary to choroidal osteoma, evidence suggests that aflibercept may offer slightly superior efficacy [15]. However, bevacizumab remains the treatment of choice when cost is a major consideration, with ranibizumab serving as an intermediate option. Importantly, the safety profiles of all three agents have been reported to be comparable [16]. In our case report, the rationale to shift from bevacizumab to aflibercept was due to the unavailability of bevacizumab. The decision to switch back to bevacizumab from aflibercept was based on observation of response on SS-OCT scans. Along with the best possible clinical response, the financial constraints of the patient favored the use of bevacizumab as the drug of choice.

Historically, laser photocoagulation [17] and photodynamic therapy [18] were employed in the management of CNV secondary to choroidal osteoma; however, the advent of intravitreal anti-VEGF therapy has largely supplanted these modalities. This was evident in our case, where treatment with bevacizumab and ranibizumab produced a favorable response, including a marked reduction in subretinal fluid clinically and as demonstrated by serial SS-OCT scans. The family and patient were counselled about the chronic nature of the disease and long-term treatment. After sub-retinal fluid has subsided, the next plan is to follow up the patient at regular intervals of three months, and plan imaging based on clinical examination, and further treatment with anti-VEGF will be commenced if there is recurrence of sub-retinal fluid.

## Conclusions

Choroidal osteoma is a rare but important cause of vision loss in children and young adults. CNV is its most frequent and vision-threatening complication. Intravitreal anti-VEGF therapy remains the gold standard, offering good functional and anatomical outcomes. This case underlines the importance of early diagnosis, multimodal imaging, and individualized long-term management. This case also demonstrates that switching between anti-VEGF agents can be considered, keeping in mind the clinical response and availability of anti-VEGF drugs. Analysis of more cases needs to be done in order to standardize treatment for CNV caused by choroidal osteoma.
